# Acute Kidney Injury and a Renal Hilar Mass in a Kidney Transplant Recipient

**DOI:** 10.34067/KID.0000000606

**Published:** 2025-02-27

**Authors:** Juan Pablo Huidobro E, Fiorella Anghileri, Patricio Downey

**Affiliations:** 1 Nephrology Department, Faculty of Medicine, School of Medicine, Pontificia Universidad Católica de Chile, Chile; 2 Transplant Institute, Red de Salud UC-Christus, Chile; 3 Kidney Medicine Transplant Department, Glickman Urological and Kidney Institute, Cleveland Clinic Foundation, Cleveland, Ohio; 4 Clinical Laboratories, Pontificia Universidad Católica de Chile, Chile

**Keywords:** AKI, cancer, kidney transplantation, lymphocytes, onconephrology

## Abstract

**Figure:**
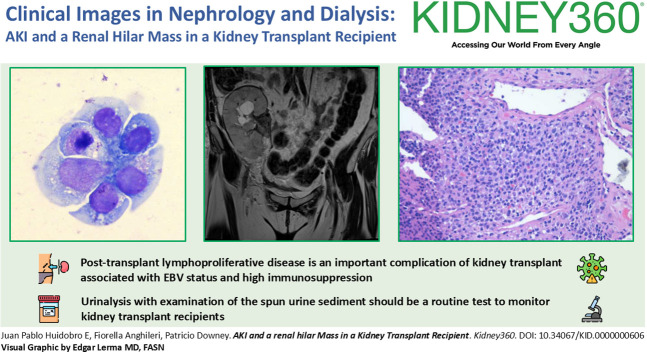

A 39-year-old woman with kidney failure secondary to lupus nephritis received an unrelated living donor kidney transplant. Calculated panel reactive antibodies were 54%, and flow cytometry cross-match was negative. She and her donor were Epstein–Barr virus (EBV) IgG positive. The patient received thymoglobulin and steroids for induction and tacrolimus, mycophenolic acid, and prednisone for chronic immunosuppression. Despite a good initial course, she developed acute antibody-mediated rejection on the second week after transplant. She was treated with plasmapheresis, immunoglobulin, and rituximab. Bortezomib was added because of lack of response. She then had a good response to treatment and serum creatinine dropped to 1.47 mg/dl.

The patient remained stable until 7 months after transplant, when urinalysis showed atypical lymphocytes (Figure 1). One month later, serum creatinine rose to 5.6 mg/dl. Allograft Doppler ultrasonography was normal. Kidney graft biopsy showed no signs of acute rejection. Because of persistence of AKI, a computed tomography scan and magnetic resonance imaging (Figure 2) were performed, showing a soft-tissue mass in the renal hilum, with moderate transplant hydronephrosis. A ureteral stent was placed, and a biopsy was obtained by ureteroscopy. Biopsy confirmed monomorphic post-transplant lymphoproliferative disorder (PTLD) (large B cells) EBV (+) with flow cytometry compatible with B-cell PTLD (Figure 3). The patient was treated with rituximab-cyclophosphamide, doxorubicin, vincristine, prednisone, and creatinine dropped to 1.34 mg/dl.

**Figure 1:**
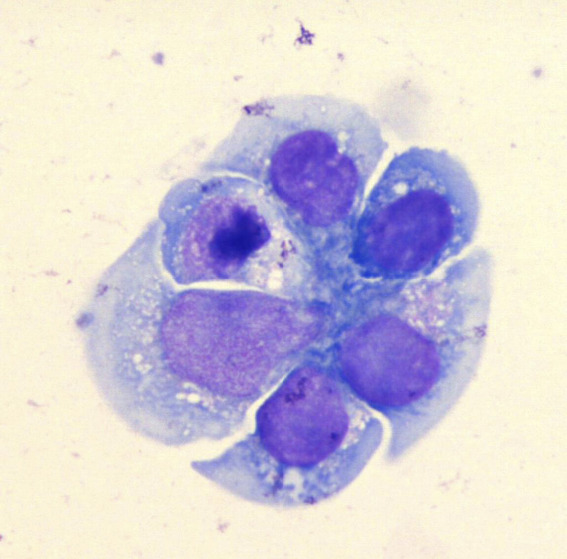
**Atypical lymphocytes in cytospun urine.** Wright staining, 100×.

**Figure 2:**
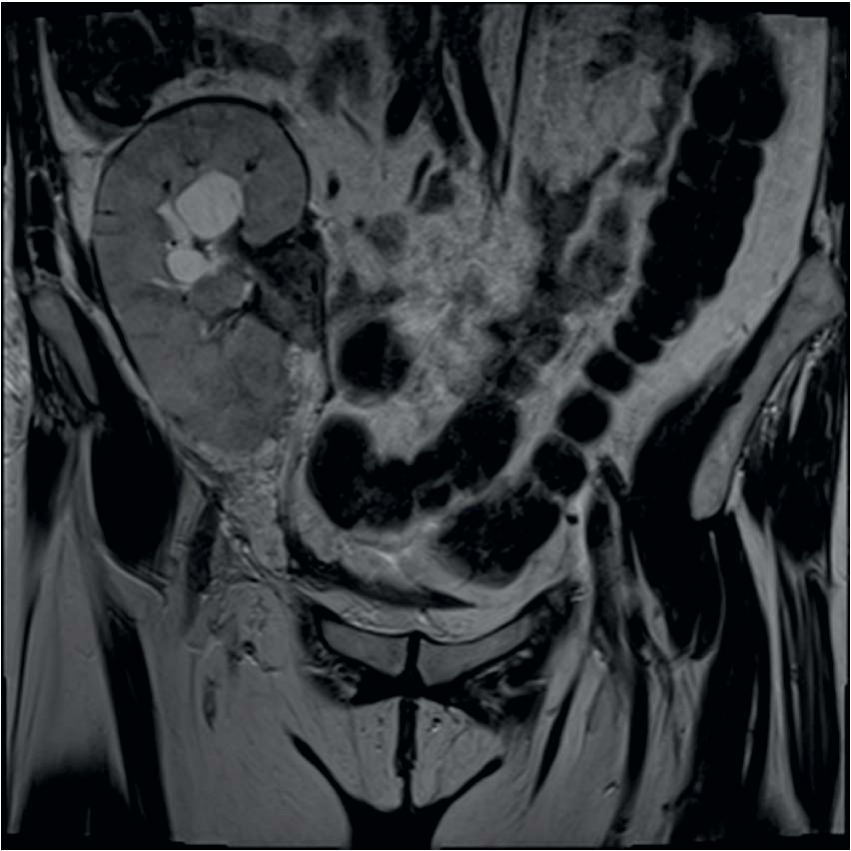
Magnetic resonance imaging, coronal T2-weighted image. Intermediate and low signal intensity mass is present in the renal hilum infiltrating the renal pelvis and surrounding normal vascular structures.

**Figure 3:**
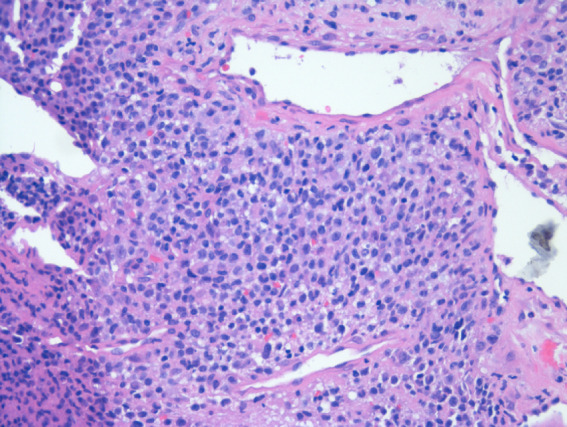
**Biopsy.** Hematoxylin-eosin staining: connective tissue fragments partially covered by urothelium, extensively infiltrated by a diffuse lymphoid proliferation and large cells with eosinophilic cytoplasm, irregular, vesicular nuclei, with one or more nucleoli and pleomorphism.

PTLD is a serious complication of kidney transplantation.^1^ Cumulative immunosuppression seems to be a major risk factor for PTLD.^2^ Clinical presentation is variable, but early-onset PTLD is more frequently EBV positive and has graft involvement.^1^ Early diagnosis of PTLD is crucial to prognosis.^3^ Urinalysis has been reported as useful to acute rejection and BK nephropathy detection,^4,5^ but it could have other potential uses. In this case, visualization of urinary atypical lymphocytes preceded clinical manifestations and imaging of urinary tract PTLD, allowing prompt diagnosis and management.

## Teaching Points

- Post-transplant lymphoproliferative disease is an important complication of kidney transplant associated with EBV status and high immunosuppression.
- Urinalysis with examination of the spun urine sediment should be a routine test to monitor kidney transplant recipients.
